# Reproducibility of ultrasound-guided attenuation parameter (UGAP) to the noninvasive evaluation of hepatic steatosis

**DOI:** 10.1038/s41598-022-06879-0

**Published:** 2022-02-21

**Authors:** Yanan Zhao, Minyue Jia, Chao Zhang, Xinxu Feng, Jifan Chen, Qunying Li, Yingying Zhang, Wen Xu, Yiping Dong, Yifan Jiang, Yajing Liu, Pintong Huang

**Affiliations:** 1 Department of Ultrasound Medicine, The Second Affiliated Hospital of Zhejiang University School of Medicine, t, 88 Jiefang Road, Shangcheng Distric, Hangzhou, 310009 Zhejiang Province China; 2 Department of Ultrasound Medicine, The Second Affiliated Hospital of Soochow University, Suzhou, 215000 China; 3 GE Healthcare Clinical Education Team, Shanghai, 200000 China

**Keywords:** Diseases, Health care

## Abstract

The aim of this study was to identify the applicability of an ultrasound-guided attenuation parameter (UGAP) for the noninvasive assessment of hepatic steatosis in clinical practice and to compare its correlation with B-mode ultrasound (US). From May to July 2021, 63 subjects with different body mass index (BMI) grades were included in the prospective study. All of them performed UGAP measurements, under different breathing manipulations, positions, diet statuses, and operators. After that, the UGAP values were compared with the visual grades of hepatic steatosis on B-mode US using a 4-point scale method. The intraclass correlation (ICC) of the UGAP values between the two radiologists was 0.862 (p < 0.001), and the ICCs of the UGAP values on the same day and different days by radiologist A were 0.899 (p < 0.001) and 0.910 (p < 0.001), respectively. There were no significant differences in UGAP values under different breathing manipulations (p > 0.05), positions (p > 0.05), or diet statuses (p = 0.300). The UGAP values in the fasting (supine position, segment V, 1) condition among the lean (BMI < 24 kg/m^2^), overweight (24 kg/m^2^ ≤ BMI < 28 kg/m^2^) and obese groups (BMI ≥ 28 kg/m^2^) were 0.60 ± 0.12, 0.66 ± 0.14, and 0.71 ± 0.11 dB/cm/MHz, respectively, with a significant difference (p = 0.006). The correlation coefficients (Rho) between the UGAP values and the visual grades of hepatic steatosis by the two reviewers were 0.845 (p < 0.001) and 0.850 (p < 0.001), corresponding to a strong relationship. Steatosis grades by reviewer 1 (p = 0.036) and reviewer 2 (p = 0.003) were significant factors determining the UGAP values according to the multivariate linear regression analysis. UGAP demonstrated excellent intraobserver and interobserver reproducibility in the assessment of hepatic steatosis. UGAP may be a promising tool in clinical practice to predict hepatic steatosis.

## Introduction

Nonalcoholic fatty liver disease (NAFLD), defined as the presence of steatosis in > 5% of hepatocytes in the absence of alcohol abuse and other known causes of liver disease^1^, is estimated to affect approximately 25% of the human population worldwide^2,3^ and may soon overtake hepatic C as the leading cause of liver transplantation^4^. It has been reported that significant steatosis can progress to nonalcoholic steatohepatitis (NASH) and clinically significant fibrosis^5,6^. Therefore, monitoring hepatic steatosis is of great significance for the early diagnosis, treatment and follow-up of NAFLD patients.

Currently, ultrasound (US) is a widely accessible imaging technique for the screening and diagnosis of liver diseases, as it is noninvasive, does not use radiation, and is economical. However, it is limited by its qualitative nature and operator dependency, and it has only modest accuracy when mild steatosis exists^7^. The controlled attenuation parameter (CAP), derived from transient elastography (TE), has been developed as an imaging tool for predicting hepatic steatosis with good diagnostic performance^8,9^. However, CAP is not an imaging modality and requires a dedicated probe, and its value is affected by the etiology and metabolic factors^10^. Recently, an alternative hepatic steatosis evaluation tool with a B-mode US system, named the ultrasound-guided attenuation parameter (UGAP, GE Healthcare), was developed that combines US image guidance and attenuation coefficient (AC) measurement for the detection of hepatic steatosis^11^.

UGAP measures the attenuation coefficient based on a tissue-mimicking reference phantom whose attenuation coefficient is known^11^. Due to the influence of the depth, frequency and inherent scattering properties of liver tissue, UGAP compensates for sound profiles by means of a fixed depth (4 cm), fixed frequency (3.5 MHz) and a known phantom as the reference. In the UGAP mode, the transmission and reception conditions are fixed to the same values as were used on the reference phantom, and the acquired echo profiles of the target are compensated by the reference data. As a result, the compensated sound profiles represent only decay caused by attenuation. If the compensated sound profile is flat, the attenuation is the same as that of the reference phantom^11,12^. Some studies have demonstrated that UGAP has good diagnostic performance for steatosis quantification in hepatitis C and NAFLD patients^11–14^. UGAP is expected to have an advantage from the perspective of real-time measurements and constant sample volumes. However, a high probability of obtaining actual applicable measurements and repeatability are necessary before UGAP can be used more widely. To the best of our knowledge, there is no research to investigate its feasibility or influencing factors in a clinical setting.

In this study, we aimed to evaluate the applicability of UGAP in different clinical settings for participants with different BMI grades. We also explored the correlation between UGAP values and the visual grades of hepatic steatosis.

## Materials and methods

### Subjects

A prospective study was conducted between May 2021 and July 2021 in the Second Affiliated Hospital of Zhejiang University School of Medicine. The study population comprised 63 consecutive subjects of different BMI grades who were approved by our institutional review board. Informed consent was obtained from all participants. All methods were performed in accordance with the Declaration of Helsinki.

The inclusion criteria were the ability to provide informed consent, age older than 18 years, and no history of liver surgery. Exclusion criteria were an inability to hold their breath for more than 3 s during the US examination; a possible other cause of chronic liver disease (i.e., excessive alcohol consumption, viral hepatitis, hepatoxic drugs, autoimmune hepatitis, etc.) and known liver lesions. Finally, 63 adults with lean weight (BMI < 24 kg/m^2^) (n = 13), overweight (24 kg/m^2^ ≤ BMI < 28 kg/m^2^) (n = 27) and obesity (BMI > 28 kg/m^2^) (n = 23) constituted the study population.

### Clinical data

Patient age, sex, height, weight and distance from the skin to the liver capsule were recorded. In this study, we defined BMI (weight in kilograms divided by the square of height in meters) of < 24 kg/m^2^ as normal/lean weigh, 24 kg/m^2^ ≤ BMI < 28 kg/m^2^ as overweight, and BMI ≥ 28 kg/m^2^ as a diagnosis of obesity on the basis of the Chinese criteria^15^.

### UGAP measurements

The patient was in the supine position with the right upper extremity extended above the head to stretch the intercostal muscles and obtain the proper scanning window during the examination. First, B-mode US images were scanned to detect if any focal liver lesion existed. Second, the UGAP mode was activated, and examinations were performed on liver segment V, inferior to the right anterior lobe, through the intercostal space, with the transducer perpendicular to the skin surface while the participant held his or her breath for 3–5 s. Third, since the depth of region of interest (ROI) is fixed from 4 to 8 cm, the operator can only move the ROI on the right and left axis to avoid bile ducts, vessels and shading artifacts (Fig. 2B). Twelve consecutive measurements on the different frames were recorded, and median and interquartile range (IQR)/median values were displayed. We defined IQR/median < 15% of twelve measurements as effective and successful measurements. The AC was calculated using the method from a prior report by Yao et al.^16^ and GE whitepaper.

### Group 1: evaluation of UGAP reproducibility in healthy subjects

**Figure 1:**
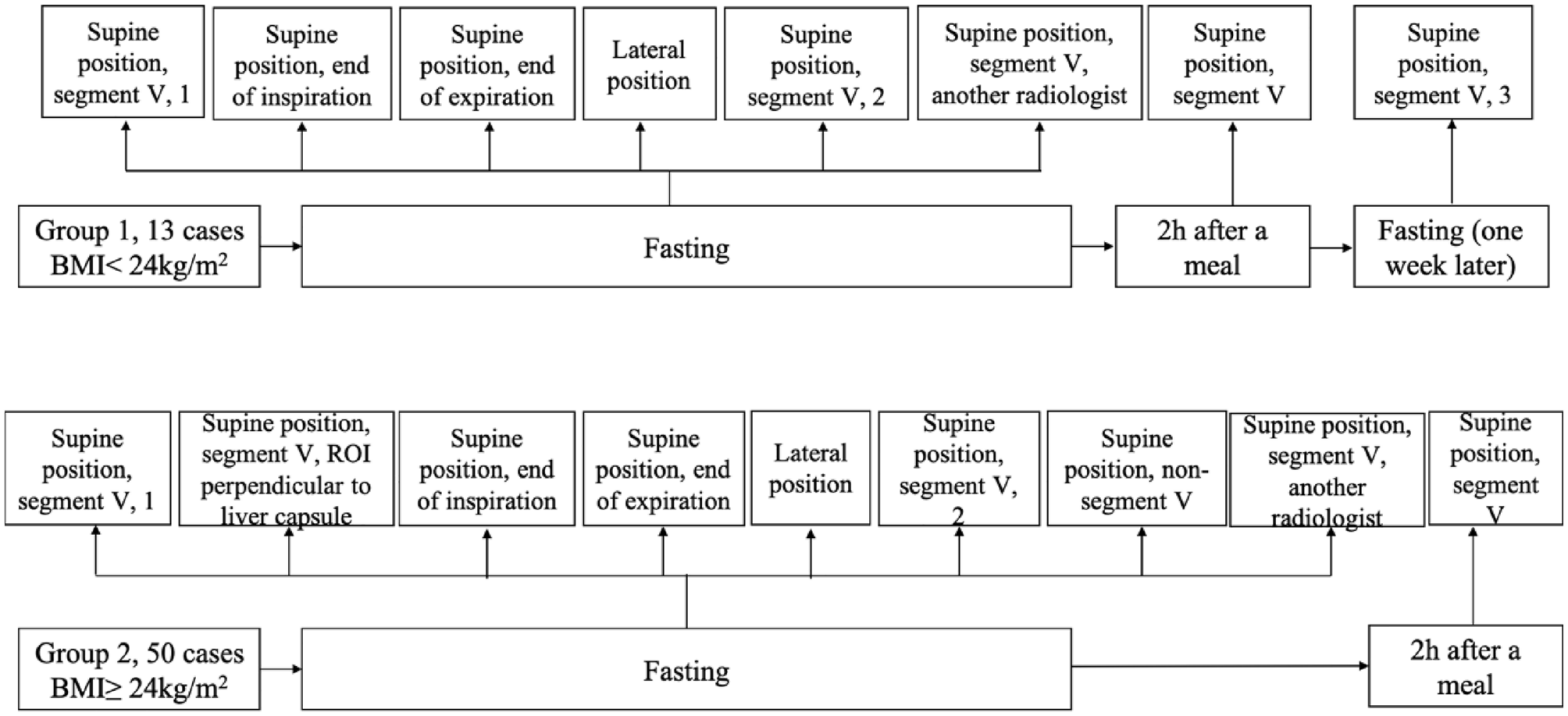
Flow chart of the study design. *BMI* body mass index.

Before the study, all 13 healthy adults with BMI less than 24 kg/m^2^ fasted for at least 8 h. UGAP measurements were performed in the supine position on the hepatic V segment during breath holding, free breathing (SP, S5, 1), deep inspiration (SP, EI), and expiration (SP, EE), and then in the lateral decubitus position during breath holding and free breathing (LP) by radiologist A. Then, radiologist B performed the same protocol in the supine position for hepatic V segment during breath holding and free breathing (SP, S5, another radiologist). After 15-min intervals and one week later, the participants were measured again by radiologist A (SP, S5, 2) (SP, S5, 3). Finally, the measurements were repeated 2 h after a meal (Fig. 1).

### Group 2: evaluation of UGAP in overweight and obese participants

We consecutively enrolled 50 subjects with a BMI greater than 24 kg/m^2^ for further evaluation. Most of the procedures were similar to those in Group 1 (Fig. 1), except that two UGAP measurements were added to different liver segments (SP, non-S5), and the ROI position was fixed perpendicular to the liver capsule (SP, S5, ROI PLC).

### Comparison of the UGAP results with the visual grades of hepatic steatosis

**Figure 2:**
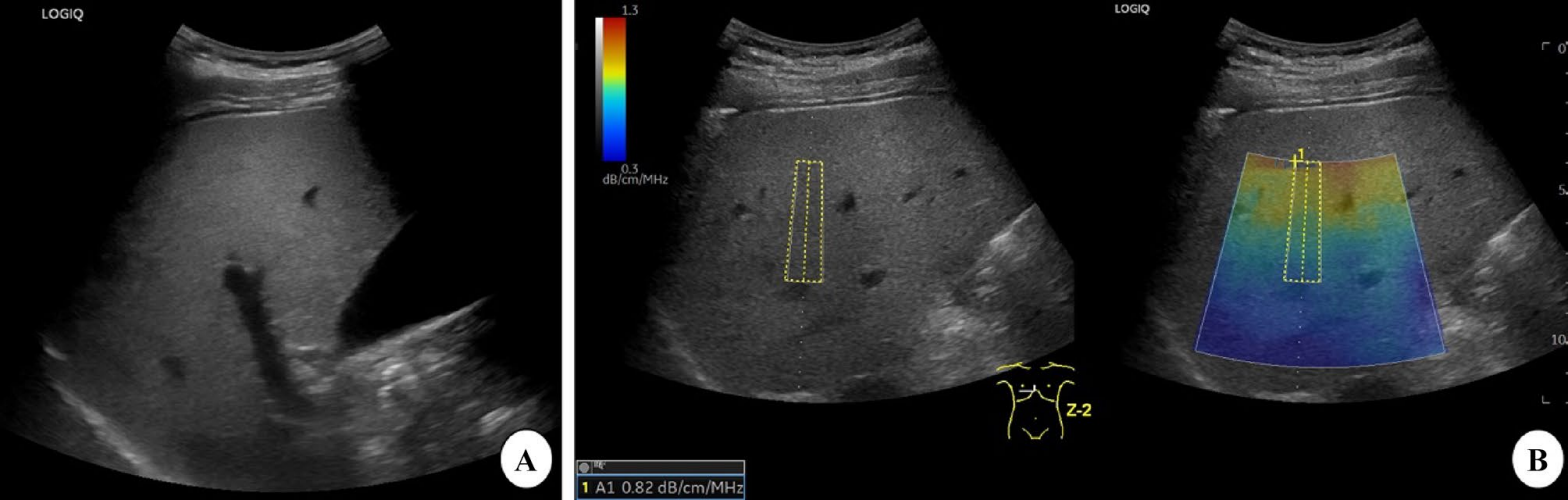
B-mode ultrasound (US) and ultrasound-guided attenuation parameter (UGAP) measurement in a 32-year-old man with hepatic steatosis. A. B-mode US imaging shows increased hepatic echogenicity with impaired visualization of periportal echogenicity and visible diaphragm echogenicity. Both reviewers 1 and 2 assessed the degree of steatosis as moderate. B. UGAP measurement was performed in the right lobe of the liver through an intercostal scan. The level of attenuation was color-coded and displayed in the region of interest (ROI), excluding the vascular structures. The ultrasound system automatically displays the attenuation coefficient (dB/cm/MHz).

The visual grade of hepatic steatosis in all 63 objects was graded on a 4-point scale through B-mode US by two independent double-blind reviewers with more than ten years of experience in abdominal ultrasonography. The diagnostic criteria were based on the following characteristics^17–19^: Grade 0, echogenicity of the liver parenchyma slightly greater than or equal to that of the renal cortex, with visible periportal and diaphragmatic echogenicity; Grade 1, increased hepatic echogenicity with visible periportal and diaphragmatic echogenicity; and Grade 2, increased hepatic echogenicity with impaired visualization of periportal echogenicity, without obscuration of the diaphragm (Fig. 2A). Grade 3, increased hepatic echogenicity with impaired visualization of periportal echogenicity and obscuration of the diaphragm.

### Statistical analysis

Statistical analysis was performed using SPSS software version 17 (IBM Corp., Armonk, NY, USA) and MedCalc software version 12.1.00 (MedCalc Software, Mariakerke, Belgium). Continuous data are expressed as the mean ± standard deviation, and count data are presented as absolute numbers or percentages. The normality of the continuous data was tested by the one-sample Kolmogorov–Smirnov test. UGAP value differences in different BMI groups were analyzed by one-way ANOVA and LSD tests for pairwise comparisons (the data satisfied the normal distribution and homogeneity of variance) and the Kruskal–Wallis H tests and Mann–Whitney U tests for pairwise comparisons (p value less than 0.017) (the data did not satisfy a normal distribution or homogeneity of variance). Intraobserver reproducibility and interobserver reproducibility of the UGAP values were assessed using intraclass correlation coefficients (ICCs). Visual grades of hepatic steatosis between the two reviewers were tested by the quadratic weighted kappa test. A paired sample t-test was used to evaluate the differences in UGAP values under different conditions. The Spearman rank correlation coefficient was used to evaluate the correlation between the UGAP values and the visual grade of hepatic steatosis. All significance tests were two-sided, and p values less than 0.05 were considered statistically significant.

### Ethical approval

This study was reviewed and approved by the Ethics Committee of Second Affiliated Hospital of Zhejiang University School of Medicine.

### Informed consent

All study participants provided informed written consent prior to study enrollment. All authors reviewed and approved the final version of the manuscript.

## Results

### Baseline characteristics

Sixty-three consecutive subjects with 13 BMI < 24 kg/m^2^ and 50 BMI > 24 kg/m^2^ were enrolled in this study. The mean (± standard deviation) values of age and BMI for all participants were 36.5 years ± 11.3 and 26.4 kg/m^2^ ± 4.1, respectively. The distance from the skin to the liver capsule was 1.9 cm ± 0.4. The baseline demographic and UGAP values under the different conditions are summarized in Table 1.

**Table 1:** Baseline characteristics of the 63 subjects.

Parameters	Group 1 (n = 13)	Group 2 (n = 50)	ALL (n = 63)	t/Z/χ^2^ value	*p* value
Age (years, mean ± SD) [range]	29.4 ± 7.7	38.5 ± 11.3	36.5 ± 11.3	− 3.256^‡^	0.001
Sex (n, male: female)	6: 7	24: 26	30: 33	0.014^ℐ^	0.905
BMI (kg/m^2^, mean ± sd) [range]	20.5 ± 2.3	28.0 ± 2.8	26.4 ± 4.1	− 8.848^†^	< 0.001
Distance from skin to liver capsule (cm, mean ± sd) [range]	1.4 ± 0.3	2.0 ± 0.4	1.9 ± 0.4	− 4.647^‡^	< 0.001
**UGAP (dB/cm/MHz)**					
Fasting (SP, S5, 1)	0.58 ± 0.09	0.69 ± 0.13	0.67 ± 0.13	− 2.923^‡^	0.003
Fasting (SP, S5, ROI PLC)	–	0.70 ± 0.13	–		
Fasting(SP, EI)	0.57 ± 0.10	0.71 ± 0.13	0.68 ± 0.13	− 3.690^†^	< 0.001
Fasting(SP, EE)	0.58 ± 0.11	0.67 ± 0.13	0.65 ± 0.13	− 2.413^‡^	0.016
Fasting(LP)	0.54 ± 0.12	0.70 ± 0.13	0.66 ± 0.15	− 3.814^†^	< 0.001
Fasting (SP, S5, 2)	0.57 ± 0.11	0.69 ± 0.12	0.67 ± 0.13	− 3.195^†^	0.002
Fasting (SP, non-S5)	–	0.69 ± 0.13	–		
Fasting (SP, S5, another radiologist)	0.56 ± 0.12	0.70 ± 0.13	0.67 ± 0.14	− 3.467^†^	0.001
2 h after a meal (SP, S5)	0.59 ± 0.10	0.67 ± 0.18	0.65 ± 0.17	− 2.584^‡^	0.010
Fasting (SP, S5, 3)	0.59 ± 0.10	–	–		

*SP* supine position, *S5* segment V, *ROI PLC* region of interest (ROI) perpendicular to liver capsule, *EI* end of inspiration, *EE* end of expiration, *LP* lateral position, *BMI* body mass index, *UGAP* ultrasound-guided attenuation parameter.

^‡^Wilcoxon Mann–Whitney rank-sum test.

^†^Paired sample T test.

^ℐ^Chi-square test.

### Interobserver and intraobserver variability of the UGAP values

The mean of the median UGAP values on the same day by radiologists A and B were 0.67 ± 0.13 and 0.67 ± 0.14 dB/cm/MHz, respectively. There was no significant difference in UGAP values between the two radiologists for all participants (t = − 0.568, p = 0.572), and the intraclass correlation coefficient (ICC) was 0.862 (p < 0.001). The means of the median UGAP values on different days by radiologist A were 0.59 ± 0.11 and 0.60 ± 0.11 dB/cm/MHz, respectively. There was no significant difference in UGAP values (z = − 0.902, p = 0.367), and the ICC was 0.910 (p < 0.001). The means of the median UGAP values between 15-min intervals by radiologist A were 0.67 ± 0.13 and 0.67 ± 0.13 dB/cm/MHz, respectively. There was no significant difference in UGAP values (t = − 0.296, p = 0.768), and the ICC was 0.899 (p < 0.001) (Table 2).

**Table 2:** Interobserver and intraobserver variability of the UGAP value assessment.

	ICC (95% CI)	*p* value
Two UGAP measurements between two radiologists	0.862 (0.783–0.914)	< 0.001*
Two UGAP measurements between one radiologist (15 min intervals)	0.899 (0.838–0.937)	< 0.001*
Two UGAP measurements between one radiologist (different days)	0.910 (0.780–0.965)	< 0.001*

*ICC* intra-class correlation coefficient, *CI* confidence interval, *UGAP* ultrasound-guided attenuation parameter.

*p < 0.05.

### Differences in UGAP values under different conditions

As the UGAP values showed great intraobserver and interobserver agreement, we compared the UGAP values in different breathing manipulations, positions and diet statuses with the first finding from the supine position and segment V (SP, S5, 1) (Table 3). As Table 3 shows, we did not find any significant differences in UGAP values under different conditions, including breathing manipulations, positions or diet statuses.

**Table 3:** Differences in the UGAP values between different breathing patterns and positions.

	t/Z value	*p* value
Fasting (SP, S5, 1) & Fasting (SP, S5, ROI PLC)	− 0.999^†^	0.323
Fasting (SP, S5, 1) & Fasting(SP, EI)	− 1.872^‡^	0.061
Fasting (SP, S5, 1) & Fasting(SP, EE)	− 1.361^‡^	0.174
Fasting (SP, S5, 1) & Fasting (SP, non-S5)	0.271^†^	0.788
Fasting (SP, S5, 1) & Fasting(LP)	0.073^†^	0.942
Fasting (SP, S5, 1) & 2 h after a meal (SP, S5)	− 1.036^‡^	0.300

UGAP values showed good intraobserver and interobserver variability, so we compared the UGAP values in the first time (Fasting [SP, S5, 1]) with the UGAP values in other conditions.

*SP* supine position, *S5* segment V, *ROI PLC* region of interest (ROI) perpendicular to liver capsule, *EI* end of inspiration, *EE* end of expiration, *LP* lateral position, *BMI* body mass index, *UGAP* ultrasound-guided attenuation parameter.

^‡^Wilcoxon Mann–Whitney rank-sum test.

^†^Paired sample T test.

### Differences in UGAP values among three groups of lean (BMI < 24 kg/m^2^), overweight (24 kg/m^2^ ≤ BMI < 28 kg/m^2^) and obese (BMI ≥ 28 kg/m^2^) participants

**Figure 3:**
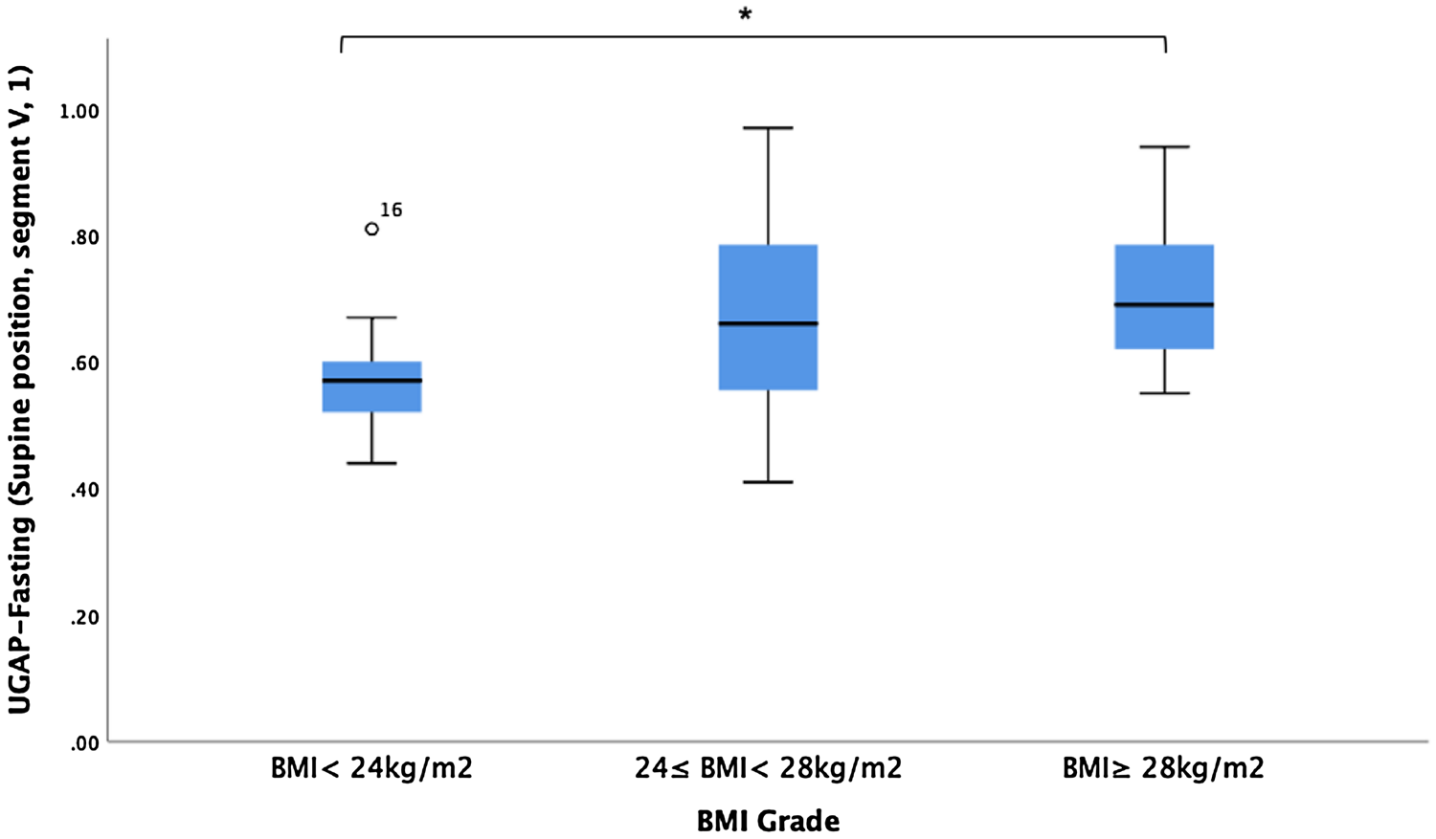
UGAP values in the fasting (SP, S5,1) condition among lean, overweight and obese participants. *p < 0.017.

The median UGAP values in the fasting (SP, S5, 1) condition among the lean (BMI < 24 kg/m^2^), overweight (24 kg/m^2^ ≤ BMI < 28 kg/m^2^) and obese groups (BMI ≥ 28 kg/m^2^) were 0.60 ± 0.12, 0.66 ± 0.14, and 0.71 ± 0.11 dB/cm/MHz, respectively, with a significant difference (p = 0.006). The UGAP values under different conditions are presented in Table 4. There were significant differences in the UGAP values among the lean, overweight and obese groups, irrespective of the breathing manipulations, positions or diet status. Further paired comparisons revealed a significant difference in the UGAP values in the fasting (SP, S5, 1) condition between lean and obese patients (Fig. 3). The distance from the skin to the liver capsule also increased with increasing BMI grades (p < 0.001).

**Table 4:** Differences in UGAP values among the three groups of BMI < 24 kg/m^2^, 24 ≤ BMI < 28 kg/m^2^ and BMI ≥ 28 kg/m^2^.

Parameters	BMI < 24 (n = 13) (dB/cm/MHz)	24 ≤ BMI < 28 (n = 27) (dB/cm/MHz)	BMI ≥ 28 (n = 23) (dB/cm/MHz)	F /Z value	*p* value
Distance from skin to liver capsule (cm)	1.48 ± 0.27	1.82 ± 0.25	2.17 ± 0.42	30.130^‡^	< 0.001*
Fasting (SP, S5, 1)	0.60 ± 0.12	0.66 ± 0.14	0.71 ± 0.11	10.249^‡^	0.006*
Fasting (SP, EI)	0.60 ± 0.15	0.68 ± 0.13	0.73 ± 0.12	7.512^§^	0.001*
Fasting (SP, EE)	0.60 ± 0.13	0.64 ± 0.14	0.70 ± 0.13	7.125^‡^	0.028*
Fasting (LP)	0.56 ± 0.14	0.64 ± 0.18	0.73 ± 0.13	9.087^§^	< 0.001*
Fasting (SP, S5, 2)	0.60 ± 0.14	0.66 ± 0.12	0.71 ± 0.12	5.865^§^	0.005*
Fasting (SP, S5, another radiologist)	0.59 ± 0.15	0.67 ± 0.12	0.72 ± 0.13	6.857^§^	0.002*
2 h after a meal (SP, S5)	0.61 ± 0.13	0.68 ± 0.13	0.65 ± 0.22	6.695^‡^	0.035*

*SP* supine position, *S5* segment V, *ROI PLC* region of interest (ROI) perpendicular to liver capsule, *EI* end of inspiration, *EE* end of expiration. *LP* lateral position, *BMI* body mass index, *UGAP* ultrasound-guided attenuation parameter.

*p < 0.05.

^‡^Kruskal–Wallis H test.

^§^One Way ANOVA.

### Correlation between UGAP values and the hepatic steatosis grades

**Figure 4:**
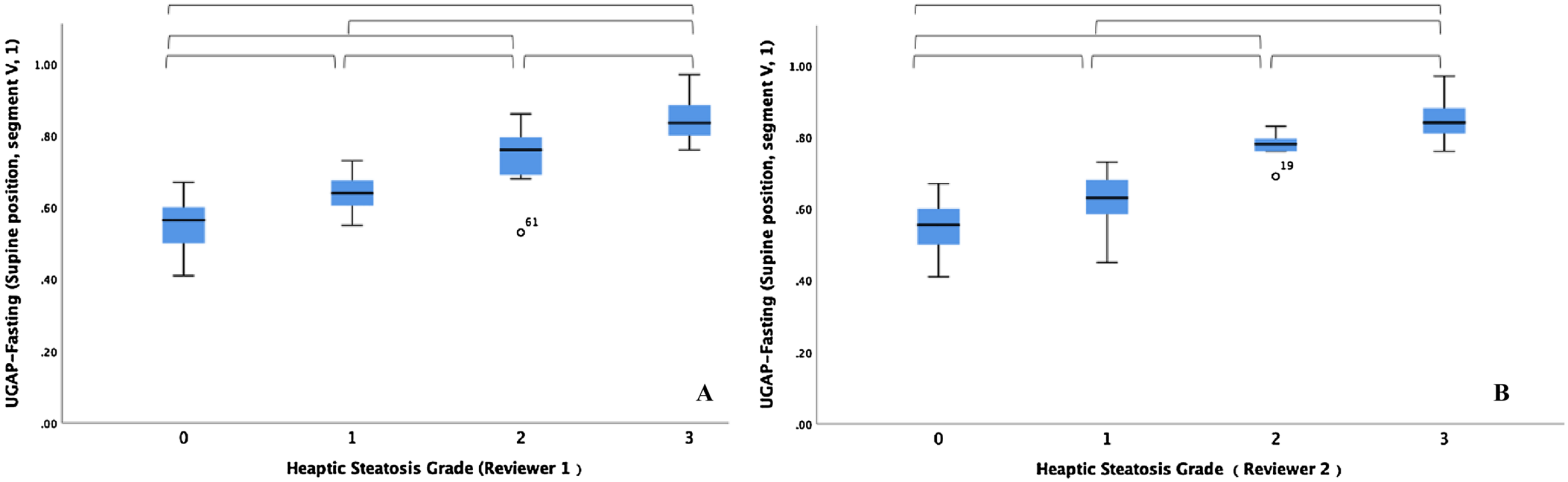
Box-and-whisker plots of the mean UGAP values according to the visual grade of hepatic steatosis of both (**A**) reviewer 1 and (**B**) reviewer 2. Median UGAP values and interquartile ranges (in parentheses) for each steatosis grade are given (**A**,**B**). The central box represents values for the lower to upper quartile (25–75 percentile). The middle line represents the median. A line extends from the minimum to the maximum value (range), excluding outlying values, which are displayed as separate points. Horizontal brackets indicate significant differences between each steatosis grade using the Mann–Whitney U tests (p < 0.0083). *UGAP* ultrasound-guided attenuation parameter.

The UGAP values and the visual grades of hepatic steatosis in all participants are presented in Table 5. The UGAP values showed a significant positive correlation with the visual grades of hepatic steatosis by both reviewer 1 (Rho, 0.845; p < 0.001) and reviewer 2 (Rho, 0.850; p < 0.001). Figure 4 shows the distribution of UGAP values over the visual grades of hepatic steatosis. The UGAP values were significantly different among all grades of hepatic steatosis (p < 0.001). The agreement rate of the two reviewers’ visual grades of hepatic steatosis reached 82.5% (52/63). Weighted kappa analysis revealed high agreement (quadratic weighted kappa, 0.930; standard error, 0.022, 95% CI, 0.887–0.974) between the visual grades assigned by the two reviewers.

**Table 5:** UGAP values according to the visual grades of hepatic steatosis in Group 1 and Group 2 (n = 63).

Visual grades of hepatic steatosis	UGAP (dB/cm/MHz)	Correlation coefficient
**Reviewer 1**		
0 (n = 24, 38.2%)	0.55 ± 0.07	Rho = 0.845*
1 (n = 15, 23.8%)	0.64 ± 0.06	
2 (n = 12, 19.0%)	0.74 ± 0.09	
3 (n = 12, 19.0%)	0.85 ± 0.06	
**Reviewer 2**		
0 (n = 20, 31.7%)	0.55 ± 0.07	Rho = 0.850*
1 (n = 23, 36.5%)	0.63 ± 0.07	
2 (n = 7, 11.1%)	0.77 ± 0.04	
3 (n = 13, 20.6%)	0.85 ± 0.06	

Values are presented as mean ± standard deviation.

*UGAP* ultrasound-guided attenuation parameter.

*p < 0.05.

### Linear regression analysis of factors affecting the UGAP value

The factors affecting the UGAP value are summarized in Table 6. We included factors in the multivariate linear regression analysis that were significantly less than 0.05 in the univariate regression analysis. According to the univariate analysis, distance from the skin to the liver capsule (p = 0.003), BMI (p = 0.001) and steatosis grades by reviewer 1 (p < 0.001) and reviewer 2 (p < 0.001) were associated with the UGAP value. However, steatosis grade by reviewer 1 (p = 0.036) and reviewer 2 (p = 0.003) were factors significantly associated with the UGAP value according to the multivariate linear regression analysis.

**Table 6:** Linear regression analysis of factors affecting the UGAP value.

Characteristics	Univariate analysis			Multivariate analysis		
	Coefficient	95% CI	*p* value	Coefficient	95% CI	*p* value
Sex	0.062	− 0.004–0.127	0.066	–	–	–
Age	0.001	− 0.001–0.004	0.322	–	–	–
Distance from skin to liver capsule	0.119	0.042–0.197	0.003	–	–	–
BMI	0.013	0.005–0.021	0.001	–	–	–
Steatosis grade (reviewer 1)	0.099	0.084–0.114	< 0.001	0.042	0.003–0.081	0.036*
Steatosis grade (reviewer 2)	0.104	0.089–0.119	< 0.001	0.064	0.023–0.104	0.003*

*BMI* body mass index, *UGAP* ultrasound-guided attenuation parameter.

*p < 0.05.

## Discussion

In our study, we demonstrated that the interobserver and intraobserver reliability of UGAP was excellent. Furthermore, the UGAP value was not affected by breathing manipulations, positions or diet statuses. There were significant differences in UGAP values among the lean, overweight and obese groups. Additionally, there were strong correlations between UGAP and the visual grades of hepatic steatosis determined by the two reviewers. The visual grades of hepatic steatosis by the two reviewers were factors significantly determining the UGAP value according to the univariate and multivariate analyses. Considering these results, we believe that UGAP may be of great clinical applicability as a screening test in patients with hepatic steatosis.

In recent years, TE or shear wave elastography (SWE) has been suggested to be a clinically useful tool for identifying advanced fibrosis in patients with NAFLD^20,21^. Instead of detecting the fat content, elastography measures the stiffness of tissue, which limits its value in the early stages of NAFLD^21^. Accordingly, UGAP based on the ultrasonic attenuation coefficient, may be more sensitive than elastography in hepatic steatosis screening. As a new technology, the sensitivity, stability and reproducibility of UGAP should be confirmed before it is widely used in clinical scenarios. Based on this consideration, we verified the intraobserver and interobserver variability of UGAP measurements, as well as whether UGAP measurements are affected by changes in breathing manipulations, positions or diet statuses. Our results demonstrated that the UGAP results had great repeatability irrespective of breathing manipulations, positions or diet statuses. Moreover, since excess body weight has a strong pathological link to NAFLD and is a critical determinant of adverse clinical outcomes^22^, the UGAP results showed a stepwise increase and significant differences among lean, overweight and obese participants, showing good feasibility for hepatic steatosis screening among all body types, which is similar to the results of another study^13^.

B-mode US is the most commonly used tool for assessing the degree of fatty liver infiltration in clinical settings^17,18^. The accuracy of B-mode US for the detection of mild steatosis (fat content > 5%) is low, with a reported sensitivity of 60.9–65%^18,23^. For the detection of moderate-severe fatty liver (> 33% steatosis), B-mode US has a performance similar to computed tomography (CT) or magnetic resonance imaging (MRI). The overall impression of B-mode US for the presence of hepatic steatosis of any degree has a sensitivity of 84.8% and a specificity of 93.6%, respectively, with an area under the ROC curve (AUC) of 0.93 [0.91–0.95], using histology as a reference standard^24^. In this study, we applied visual grades of hepatic steatosis using some US features that include liver brightness, visualization of intrahepatic ducts and diaphragm visibility. The results showed that the grades were in high agreement between the two reviewers, and the agreement rate reached 82.5% (52/63). Eleven cases of inconsistency mainly occurred in the assessment of no or mild steatosis, which was similar to previous results that the sensitivity of US decreases at lower grades of steatosis^17^. UGAP values showed a distinguished, positive correlation with the visual grades of hepatic steatosis by both reviewers and showed significant differences in different grades of hepatic steatosis, which indicates that UGAP will be a promising technique in the diagnosis of fatty liver in the future.

The CAP technique is another attenuation measurement tool, and there have been many reports on its usefulness in quantifying hepatic steatosis^25,26^. However, despite the high accuracy of CAP in detecting hepatic steatosis, it has a high failure rate in obese patients and no image guide for ROI placement, which may be vulnerable to complex wave patterns such as reflections and refractions^27^, thus limiting its ability in a significant proportion of obese patients^8,10^. An additional XL probe is necessary to reduce the failure rate but it increases the cost^28^. In the present study, there were no failed cases for the UGAP measurements, and thick subcutaneous adipose tissue could be avoided through ROI positioning guided by US images on homogenous hepatic parenchyma. Furthermore, the UGAP software is installed on the US system and the measurement can be performed with a single probe, which is more cost-efficient than CAP, which requires specialized devices and specific probes depending on the patient’s body type^29^. It has also been reported that UGAP measurements are better than CAP for detecting hepatic steatosis in patients undergoing liver biopsy^11^. Moreover, liver stiffness does not affect UGAP measurement in the evaluation of hepatic steatosis^30^, while CAP values are thought to be influenced by hepatic fibrosis^8^. Unfortunately, a comparison between UGAP and CAP was not conducted here due to the unavailability and cost of CAP.

In the present study, we also analyzed the factors that affect the UGAP values. In multiple regression analysis, visual grades of hepatic steatosis by two reviewers were the factors that significantly influenced the UGAP values, which is consistent with a previous study^11^. Although the visual grade of steatosis is not the gold standard reference, this also implied to a certain extent that hepatic steatosis is a strong histopathological independent factor for UGAP. Additional studies are warranted to include more parameters to explore other factors correlated with UGAP values.

There are several limitations of our study. First, our sample size was small, and additional large cohort studies are required to check our preliminary results. Second, we compared UGAP with US images instead of liver biopsy results. These results may not truly reflect the grade of steatosis. However, our study’s aim was to identify the applicability of UGAP as a screening tool in clinical practice rather than to evaluate the diagnostic performance of UGAP. Additional prospective studies will be designed to evaluate UGAP’s accuracy for fatty liver diagnosis, especially in no or mild hepatic steatosis using pathologic assessment or magnetic resonance imaging derived proton density fat fraction (MRI-PDFF) as a reference standard. Third, interobserver reproducibility on different days was only performed in Group 1 but not in all participants. However, excellent interobserver reproducibility on two different days was demonstrated in Group 1, so the test–retest reproducibility was only tested on the same day with a 15-min interval in Group 2. Last, this study did not conduct further research on metabolic-associated fatty liver disease (MAFLD) since NAFLD was renamed MAFLD in 2020 in an international expert consensus statement^22^. However, considering the lack of epidemiology and remaining issues to be solved, we considered it was not an appropriate time to complicate our research, and we have not performed blood tests on our participants, which may also lead to selection bias in the inclusion criteria. Future research should focus on a more careful design for MAFLD patients.

## Conclusion

In conclusion, UGAP demonstrated excellent intraobserver and interobserver reproducibility in the assessment of hepatic steatosis, as well as a strong correlation with the visual grade of steatosis, irrespective of breathing manipulations, positions or diet statuses. Therefore, UGAP has good feasibility and clinical applicability and is worthy of further research and application.
